# The association of genetic factors with serum calretinin levels in asbestos-related diseases

**DOI:** 10.2478/raon-2023-0061

**Published:** 2023-11-30

**Authors:** Cita Zupanc, Alenka Franko, Danijela Strbac, Viljem Kovac, Vita Dolzan, Katja Goricar

**Affiliations:** Military Medical Unit-Slovenian Army, Ljubljana, Slovenia; University of Ljubljana, Faculty of Medicine, Ljubljana, Slovenia; University Medical Centre Ljubljana, Clinical Institute of Occupational Medicine, Ljubljana, Slovenia; Institute of Oncology Ljubljana, Ljubljana, Slovenia; University of Ljubljana, Faculty of Medicine, Institute of Biochemistry and Molecular Genetics, Pharmacogenetics Laboratory, Ljubljana, Slovenia

**Keywords:** malignant mesothelioma, calretinin, CALB2, asbestos-related disease, polymorphism

## Abstract

**Background:**

Asbestos exposure is associated with different asbestos-related diseases, including malignant mesothelioma (MM). MM diagnosis is confirmed with immunohistochemical analysis of several markers, including calretinin. Increased circulating calretinin was also observed in MM. The aim of the study was to determine if *CALB2* polymorphisms or polymorphisms in genes that can regulate calretinin expression are associated with serum calretinin levels or MM susceptibility.

**Subjects and methods:**

The study included 288 MM patients and 616 occupationally asbestos-exposed subjects without MM (153 with asbestosis, 380 with pleural plaques and 83 without asbestos-related disease). Subjects were genotyped for seven polymorphisms in *CALB2*, *E2F2*, *MIR335*, *NRF1* and *SEPTIN7* genes using competitive allele-specific polymerase chain reaction (PCR). Serum calretinin was determined with ELISA in 545 subjects. Nonparametric tests, logistic regression and receiver operating characteristic (ROC) curve analysis were used for statistical analysis.

**Results:**

Carriers of at least one polymorphic *CALB2* rs889704 allele had lower calretinin levels (P = 0.036). Carriers of two polymorphic *MIR335* rs3807348 alleles had higher calretinin (P = 0.027), while carriers of at least one polymorphic *NRF1* rs13241028 allele had lower calretinin levels (P = 0.034) in subjects without MM. Carriers of two polymorphic *E2F2* rs2075995 alleles were less likely to develop MM (odds ratio [OR] = 0.64, 95% confidence interval [CI] = 0.43-0.96, P = 0.032), but the association was no longer significant after adjustment for age (P = 0.093). Optimal serum calretinin cut-off values differentiating MM patients from other subjects differed according to *CALB2*, *NRF1*, *E2F2*, and *MIR335* genotypes.

**Conclusions:**

The results of presented study suggest that genetic variability could influence serum calretinin levels. These findings could contribute to a better understanding of calretinin regulation and potentially to earlier MM diagnosis.

## Introduction

Prolonged asbestos exposure can lead to occurrence of different asbestos-related diseases, including pleural plaques and asbestosis, as well as several cancers. Use and production of asbestos was largely banned after it was classified as a carcinogen, but it is still legally used in mostly developing countries and it can still be found in the environment.^1,2^ Asbestos-related diseases often occur long after initial asbestos exposure and their incidence continues to rise.^1^

The most problematic asbestos-related disease is malignant mesothelioma (MM), a rare but very aggressive cancer. However, only a minority of asbestos-exposed people develops MM. Other factors, such as genetic variability may contribute to carcinogenesis and development of MM.^3^ Among asbestos-exposed workers, several familial cases of MM were described, emphasizing that genetic factors could contribute to MM development.^4^ In recent years, germline BRCA1-associated protein 1 (BAP1) mutations were shown to predispose to the development of MM and other cancers. Additionally, studies suggest that numerous chromosomal deletions can accumulate in most MM cases, usually associated with the loss or inactivation of tumor suppressor genes.^5,6^ Despite advances in treatment, prognosis and survival of MM patients remain poor.^7,8^ Therefore, MM diagnosis and treatment have become increasingly focused on molecular mechanisms.^9^

To confirm MM diagnosis, several tumor markers are routinely analysed using immunohistochemical staining.^10^ One of the established immunohistochemical markers is calretinin^10^, a calcium binding protein and calcium sensor crucial for neuron function that is also expressed on mesothelial cells.^11^ It has been shown to affect mesothelial cell proliferation and migration and epithelialto-mesenchymal transition. It was also associated with focal adhesion kinase signaling pathway and signaling pathways associated with response to asbestos.^12^ Calretinin is encoded by the *CALB2* gene.^13^

As MM diagnosis is usually made when the disease is already advanced, blood-based biomarkers such as mesothelin and fibulin-3 that would enable an earlier diagnosis and better prognosis of MM are extensively studied.^14,15^ Recently, calretinin was also proposed as a soluble biomarker in MM, as increased plasma or serum levels were observed in MM patients compared to subjects with other asbestos-related diseases or healthy controls.^8,16,17,18^ However, interindividual variability limits the sensitivity and specificity of calretinin as a diagnostic biomarker and several clinical characteristics were previously associated with soluble calretinin levels.^19^ Low tumor calretinin expression was associated with lower protein concentration in the bloodstream, but there was no clear correlation with tumor size.^20^ Higher calretinin concentrations were observed in patients with epithelioid or biphasic MM compared to patients with sarcomatoid MM.^8,20,21^ Calretinin levels were also higher in women compared to men and in subjects with renal dysfunction.^22^

Molecular mechanisms regulating calretinin expression in various tissues or in cancer could also contribute to interindividual variability of serum calretinin concentration, but the knowledge of these processes is limited.^23^ Calretinin expression may be affected by several factors, including transcription factors or miRNAs. Among transcription factors, calretinin expression was found to be influenced by septin 7, E2F transcription factor 2 (E2F2) and nuclear respiratory factor 1 (NRF-1) in previous studies.^23,24^ Additionally, miR-335-5p was proposed as a regulator of *CALB2* expression^25^ and miR-30e-5p was negatively correlated with the calretinin expression in pleural MM patient samples.^26^ Gene expression can also be modified by genetic variability in the promoter 5′ untranslated region (UTR) of the gene affecting binding of transcription factors, or genetic variability in the 3′ UTR affecting miRNA binding. Polymorphisms in genes coding for miRNAs or transcription factors involved in calretinin regulation could also influence calretinin expression. In previous studies, genetic factors affecting expression and circulating levels of other important MM biomarkers such as mesothelin have already been identified.^27,28,29^ On the other hand, very little is known about the role of single nucleotide polymorphisms (SNPs) in the *CALB2* gene. An intronic polymorphism in *CALB2* gene was previously proposed as a risk factor for colon cancer.30 To date, no studies have been performed to evaluate if genetic factors influence calretinin expression or if they could modify susceptibility to develop asbestos-related diseases.

Our aim was to determine whether genetic polymorphisms in the *CALB2* gene and in the genes coding for miRNA and transcription factors regulating calretinin expression are associated with MM susceptibility or serum calretinin levels in patients with asbestos-related diseases.

## Subjects and methods

### Study population

Our retrospective study included patients with MM, subjects with asbestosis, subjects with pleural plaques, and subjects that were occupationally exposed to asbestos but, did not develop any asbestos-related disease.

Patients with MM were treated at the Institute of Oncology Ljubljana between November 2001 and March 2019. The diagnosis of pleural or peritoneal MM was performed by thoracoscopy or laparoscopy, respectively, and confirmed histologically by an experienced pathologist, mostly in others tertiary institutions in Slovenia. Stage of MM was determined using the TNM staging system for pleural MM. Performance status of MM patients was determined using Eastern Cooperative Oncology Group (ECOG) scores.

Subjects with asbestosis, subjects with pleural plaques and asbestos-exposed subjects who did not develop any asbestos-related disease were selected from a cohort of occupationally exposed workers who were evaluated by the State Board for the Recognition of Occupational Asbestos Diseases at the Clinical Institute of Occupational, Traffic and Sports Medicine in Ljubljana between September 1998 and April 2007. The diagnosis of asbestos-related diseases was based on the Helsinki Criteria for Diagnosis and Attribution of Asbestos Diseases^31^ and the American Thoracic Society recommendations.^32^ Follow-up was performed for all subjects in 2018 to confirm they did not develop any other asbestos-related disease.

For all subjects, data on demographic (sex, age, smoking) and clinical characteristics were obtained from the medical records or during an interview. All participants provided written informed consent. The study has been approved by the National Medical Ethics Committee of the Republic of Slovenia (31/07/04, 39/04/06 and 41/02/09) and was carried out according to the Declaration of Helsinki.

### Bioinformatic analysis

Using bioinformatic analysis, we identified common SNPs that could affect calretinin expression: SNPs in the 5′ UTR and 3′ UTR of the calretinin gene (*CALB2*) and SNPs in the genes coding for miRNAs and transcription factors involved in the regulation of calretinin expression. Experimentally confirmed miRNAs and transcription factors were selected using miRTarBase^33^ and literature screening.

Using LD Tag SNP Selection tool^34^ and dbSNP database^35^, we identified all SNPs in 5′ UTR, 3′ UTR and near gene regions (± 1000 base pairs) of *CALB2* gene and all SNPs in 5′ UTR, 3′ UTR and coding regions of transcription factor coding genes with minor allele frequency (MAF) in European populations above 5%. Additionally, available literature was screened for SNPs in miRNA coding genes.^36^ *In silico* predicted function of SNPs was assessed using SNP Function Prediction tool^34^ as well as HaploReg v4.1^37^ and GTEx38 for SNPs in regulatory regions. Linkage disequilibrium (LD) between SNPs in one gene was evaluated using LD link tool.^39^ For genotyping, we selected only SNPs with *in silico* predicted functional role (non-synonymous SNPs, SNPs that influence transcription factor or miRNA binding or SNPs that influence splicing). If more SNPs within one gene were in high LD (R^2^ > 0.8), only one SNP was selected for genotyping analyses.

### DNA extraction and genotyping

Genomic DNA was extracted from peripheral venous blood samples using Qiagen FlexiGene Kit (Qiagen, Hilden, Germany) according to the manufacturer's instructions. For a subset of subjects that did not develop any asbestos-related disease, DNA was extracted from capillary blood samples collected on Whatman FTA cards using MagMax^TM^ DNA Multi-Sample Kit (Applied Biosystems, Foster City, California, USA). The genotyping of all selected SNPs was carried out using a fluorescence-based competitive allele-specific polymerase chain reaction (KASP) assay, according to the manufacturer's instructions (LGC Genomics, UK). For all SNPs, 15% of samples were genotyped in duplicates. Genotyping quality control criteria were 100% duplicate call rate and 95% SNP-wise call rate.

### Measurement of serum calretinin

Serum samples were collected at diagnosis for MM patients and at inclusion in the study for all other subjects. Samples were prepared within 6 hours of blood collection and stored at −20°C. Serum calretinin levels were determined using a commercially available enzyme-linked immunosorbent Calretinin ELISA assay (DLD Diagnostika GmbH, Germany) according to the manufacturer's instructions as previously described.^8,16,21^

### Statistical Analysis

Continuous and categorical variables were described using median with interquartile range and frequencies, respectively. Nonparametric Mann-Whitney test or Kruskal-Wallis test with post hoc Bonferroni corrections for pairwise comparisons were used to compare the distribution of continuous variables. Chi square test was used to compare the distribution of categorical variables among different groups and to evaluate deviation from Hardy-Weinberg equilibrium. For all investigated SNPs, both additive and dominant models were used in the analysis. Univariate and multivariate logistic regression was used to compare genotype frequencies between groups and to determine odds ratios (ORs) and 95% confidence intervals (CIs). Demographic and clinical parameters, significantly associated with asbestos-related disease susceptibility in univariate analysis, were used for adjustment in multivariate models. Receiver operating characteristic (ROC) curve analysis was used to determine area under the curve (AUC), sensitivity and specificity. Cut-off values were determined as the values with the highest sum of sensitivity and specificity. All statistical tests were two-sided and the level of significance was set at 0.05. The statistical analyses were carried out by using IBM SPSS Statistics version 27.0 (IBM Corporation, Armonk, NY, USA). To assess the combined effect of all *CALB2* SNPs, we reconstructed haplotypes using Thesias software.^40^ Haplotypes with predicted frequency above 0.04 were included in the analysis and the most common haplotype was used as a reference.

## Results

### Subjects’ characteristics

Among 904 subjects included in our study, 288 (31.9%) had MM. Among 616 non-MM subjects that were occupationally exposed to asbestos, 153 subjects had asbestosis, 380 subjects had pleural plaques and 83 did not develop any asbestos-related disease. Characteristics of each subject group are presented in Table 1. Patients with MM were older than all other groups (P < 0.001), but there were no significant differences regarding sex (P = 0.180) and smoking (P = 0.205).

**TABLE 1.** Clinical characteristics of the subjects included in the study

**Characteristic**	**Category/unit**	**No disease (N = 83)**	**Pleural plaques (N = 380)**	**Asbestosis (N = 153)**	**MM (N = 288)**	**P**
Sex	Male, N (%)	61 (73.5)	262 (68.9)	119 (77.8)	213 (74.0)	0.180^1^
	Female, N (%)	22 (26.5)	118 (31.1)	34 (22.2)	75 (26.0)	
Age	Median (25%–75%)	53.4 (48.5–59.2)	54.8 (48.8–62.7)	59.4 (51.3–66.1)	66.0 (59–73)	< 0.001^2^
Smoking	No, N (%)	46 (55.4)	187 (49.2)	74 (48.4)	158 (56.4) [8]	0.205^1^
	Yes, N (%)	37 (44.6)	193 (50.8)	79 (51.6)	122 (43.6)	

1 calculated using chi-square test;

2 calculated using Kruskal-Wallis test.

Number of missing data is presented in [] brackets.

MM = malignant mesothelioma

Among patients with MM, 217 (75.3%) patients had epithelioid histological type, 26 (9.0%) patients had biphasic type, and 26 (9.0%) patients had sarcomatoid type, while histological type could not be determined in 19 (6.6%) patients. According to cancer stage, 19 (6.6%) patients had stage 1 MM, 63 (22.0%) patients had stage 2 MM, 85 (29.6%) patients had stage 3 MM, and 87 (30.3%) patients had stage 4 MM, while no data were available for one patient. Additionally, 33 (11.5%) patients had peritoneal MM. Regarding ECOG performance status, 18 patients (6.3%) had score 0, 142 (49.5%) score 1, 110 (38.3%) score 2 and 17 (5.9%) score 3, while no data was available for one patient.

### Bioinformatic analysis

Based on available literature and publicly available databases, we identified genes and SNPs that could influence calretinin expression and serum levels: SNPs in 5′ and 3′ UTR of *CALB2* gene and SNPs in genes coding for transcription factors and miRNAs associated with calretinin expression. Three miRNAs were experimentally associated with regulation of *CALB2* expression: hsa-miR-9, hsamiR-30e and hsa-miR-335-5p^26^ but common SNPs were only described in *MIR335* gene. Additionally, three transcription factors were experimentally associated with regulation of *CALB2* expression: E2F transcription factor 2 (*E2F2*), nuclear respiratory factor 1 (*NRF1*), and septin 7 (*SEPTIN7*).^23,24^

In total, seven SNPs fulfilling all inclusion criteria were included in the study: *CALB2* rs1862818, *CALB2* rs889704, *CALB2* rs8063760, *E2F2* rs2075995, *MIR335* rs3807348, *NRF1* rs13241028, and *SEPTIN7* rs3801339. Their role, predicted function and genotype frequencies in the whole study group as well as minor allele frequency and agreement with Hardy-Weinberg equilibrium (HWE) in controls are presented in Table 2. All SNPs were in agreement with HWE in controls without asbestos related diseases and variant allele frequencies ranged between 14 and 63%.

**TABLE 2.** Genotype frequencies of investigated single nucleotide polymorphisms (SNPs) in the whole study group, their variant allele frequency (VAF) and agreement with Hardy-Weinberg equilibrium (HWE) in subjects without any asbestos-related disease (controls)

**Gene**	**SNP**	**Nucleotide or amino acid change**	**Predicted function**	**Genotype**	**N (%)**	**VAF (controls)**	**pHWE (controls)**
***CALB2***	rs1862818	c.-828C>T	May influence transcription factor binding, may alter chromatin states and regulatory motifs	CC	479 (53.0)	0.27	0.617
				CT	346 (38.3)		
				TT	79 (8.7)		
***CALB2***	rs889704	c.-634C>A	May influence transcription factor binding, may alter chromatin states and regulatory motifs	CC	708 (78.4) [1]	0.14	0.814
				CA	182 (20.2)		
				AA	13 (1.4)		
***CALB2***	rs8063760	c.*138T>C	May influence miRNA binding, may alter regulatory motifs	CC	527 (58.4) [2]	0.23	0.322
				CT	319 (35.4)		
				TT	56 (6.2)		
***E2F2***	rs2075995	c.678C>A, p.Gln226His	Nonsynonymous, may influence splicing	CC	187 (20.7)	0.61	0.209
				CA	468 (51.8)		
				AA	249 (27.5)		
***MIR335***	rs3807348	g.130496266G>A	Downstream transcript variant, may influence transcription factor binding	GG	228 (25.3) [3]	0.49	0.376
				GA	446 (49.5)		
				AA	227 (25.2)		
***NRF1***	rs13241028	c.*1321T>C	May influence miRNA binding	TT	547 (60.5)	0.22	0.061
				TC	313 (34.6)		
				CC	44 (4.9)		
***SEPTIN7***	rs3801339	c.1168-4451T>C	Genic downstream transcript variant^1^	TT	164 (18.1)	0.63	0.187
				TC	401 (44.4)		
				CC	339 (37.5)		

1 previously classified as a nonsynonymous variant.

Number of missing data is presented in [] brackets.

A = adenine; C = cytosine; G = guanine; SNP = single nucleotide polymorphisms; T = thymine

### Association of selected SNPs with MM susceptibility

In the whole study group, we evaluated if selected polymorphisms were associated with MM susceptibility. Genotype frequencies in MM patients and subjects without MM and are presented in Table 3. Carriers of two polymorphic *E2F2* rs2075995 alleles were less likely to develop MM (OR = 0.64, 95% CI = 0.43–0.96, P = 0.032), but the association was no longer significant after adjustment for age (OR = 0.68, 95% CI = 0.44–1.07, P = 0.093). No other SNP was significantly associated with MM susceptibility (Table 3). Additionally, we also compared MM patients to other subject groups separately. Genotype frequencies of SNPs among subjects with asbestosis, subjects with pleural plaques and subjects without asbestos-related diseases, are presented in Supplementary Table 1. When comparing MM patients with subjects without any asbestos-related disease, carriers of two polymorphic *E2F2* rs2075995 alleles were less likely to develop MM (OR = 0.35, 95% CI = 0.16–0.78, P = 0.010), even after adjustment for age (OR = 0.35, 95% CI = 0.14–0.84, P = 0.019). The association with MM susceptibility was significant also in the dominant model, both in univariate (OR = 0.43, 95% CI = 0.21–0.87, P = 0.019) and multivariate (OR = 0.43, 95% CI = 0.19–0.94, P = 0.033) analysis. Compared to subjects with asbestosis, carriers of two polymorphic *MIR335* rs3807348 alleles were more likely to develop MM (OR = 1.82, 95% CI = 1.05–3.16, P = 0.033), even after adjustment for age (OR = 0.35, 95% CI = 1.10–3.50, P = 0.022). After adjustment for age, the association with MM susceptibility was significant also in the dominant model (OR = 1.62, 95% CI = 1.03–2.55, P = 0.037). None of the other SNPs was significantly associated with MM susceptibility (Supplementary Table 2).

**TABLE 3.** Association of investigated single nucleotide polymorphisms (SNPs) with malignant mesothelioma (MM) susceptibility

**SNP**	**Genotype**	**Subjects without MM (N = 616) N (%)**	**MM patients (N = 288) N (%)**	**OR (95% CI)**	**P**	**OR (95% CI)_adj_**	**P_adj_**
***CALB2* rs1862818**	CC	340 (55.2)	139 (48.3)	Reference		Reference	
	CT	226 (36.7)	120 (41.7)	1.30 (0.97–1.75)	0.084	1.35 (0.97–1.87)	0.073
	TT	50 (8.1)	29 (10.1)	1.42 (0.86–2.34)	0.169	1.34 (0.77–2.32)	0.299
	CT+TT	276 (44.8)	149 (51.7)	1.32 (1.00–1.75)	0.052	1.35 (0.99–1.83)	0.059
***CALB2* rs889704**	CC	485 (78.9) [1]	223 (77.4)	Reference		Reference	
	CA	121 (19.7)	61 (21.2)	1.10 (0.78–1.55)	0.602	1.03 (0.70–1.51)	0.899
	AA	9 (1.5)	4 (1.4)	0.97 (0.29–3.17)	0.955	0.55 (0.15–1.94)	0.349
	CA+AA	130 (21.1)	65 (22.6)	1.09 (0.78–1.52)	0.626	0.98 (0.67–1.42)	0.912
***CALB2* rs8063760**	CC	352 (57.3) [2]	175 (60.8)	Reference		Reference	
	CT	222 (36.2)	97 (33.7)	0.88 (0.65–1.19)	0.398	0.91 (0.66–1.26)	0.576
	TT	40 (6.5)	16 (5.6)	0.80 (0.44–1.48)	0.483	0.82 (0.42–1.60)	0.554
	CT+TT	262 (42.7)	113 (39.2)	0.87 (0.65–1.15)	0.329	0.90 (0.65–1.23)	0.493
***E2F2* rs2075995**	CC	117 (19.0)	70 (24.3)	Reference		Reference	
	CA	319 (51.8)	149 (51.7)	0.78 (0.55–1.11)	0.171	0.83 (0.56–1.23)	0.349
	AA	180 (29.2)	69 (24.0)	0.64 (0.43–0.96)	**0.032**	0.68 (0.44–1.07)	0.093
	CA+AA	499 (81.0)	218 (75.7)	0.73 (0.52–1.02)	0.067	0.78 (0.53–1.13)	0.182
***MIR335* rs3807348**	GG	158 (25.8) [3]	70 (24.3)	Reference		Reference	
	GA	307 (50.1)	139 (48.3)	1.02 (0.72–1.44)	0.902	1.00 (0.68–1.46)	0.98
	AA	148 (24.1)	79 (27.4)	1.20 (0.81–1.78)	0.352	1.22 (0.79–1.87)	0.376
	GA+AA	455 (74.2)	218 (75.7)	1.08 (0.78–1.50)	0.636	1.07 (0.75–1.52)	0.724
***NRF1* rs13241028**	TT	374 (60.7)	173 (60.1)	Reference		Reference	
	TC	210 (34.1)	103 (35.8)	1.06 (0.79–1.43)	0.699	1.08 (0.78–1.50)	0.636
	CC	32 (5.2)	12 (4.2)	0.81 (0.41–1.61)	0.550	0.92 (0.44–1.93)	0.823
	TC+CC	242 (39.3)	115 (39.9)	1.03 (0.77–1.37)	0.853	1.06 (0.78–1.45)	0.711
***SEPTIN7* rs3801339**	TT	109 (17.7)	55 (19.1)	Reference		Reference	
	TC	266 (43.2)	135 (46.9)	1.01 (0.68–1.48)	0.976	1.05 (0.69–1.61)	0.815
	CC	241 (39.1)	98 (34.0)	0.81 (0.54–1.20)	0.291	0.76 (0.49–1.18)	0.218
	TC+CC	507 (82.3)	233 (80.9)	0.91 (0.64–1.30)	0.610	0.91 (0.61–1.35)	0.627

Number of missing data is presented in [] brackets.

A = adenine; Adj = adjusted for age; C = cytosine; CI = confidence interval; G = guanine; OR = odds ratio; T= thymine

### Association of selected SNPs with serum calretinin levels

Serum calretinin concentration was determined in 545 subjects. Calretinin concentration significantly differed among subject groups (P < 0.001): MM patients (N = 163) had median calretinin concentration 0.52 (0.23–1.43) ng/ml, subjects with asbestosis (N = 117) 0.13 (0.08–0.20) ng/ml, subjects with pleural plaques (N = 195) 0.18 (0.12–0.25) ng/ml and subjects without disease (N = 70) 0.12 (0.07–0.19) ng/ml.

**FIGURE 1.**
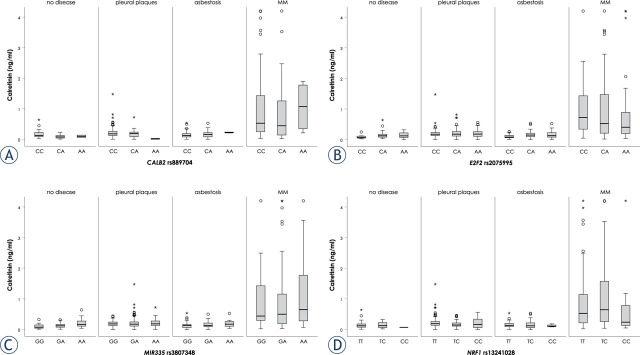
Association of selected single nucleotide polymorphisms (SNPs) with serum calretinin concentration: *CALB2* rs889704 **(A)**, *E2F2* rs2075995 **(B)**, *MIR335* rs3807348 **(C)**, *NRF1* rs13241028 **(D)**.

The association of selected SNPs with serum calretinin concentration is presented in Table 4 and Figure 1. In all subjects, carriers of at least one polymorphic *CALB2* rs889704 A allele had lower calretinin than carriers of two wild-type alleles in the dominant model (P = 0.036), but no significant differences were observed if subjects without MM and MM patients were evaluated separately (P = 0.069 and 0.441, respectively). In the group of subjects without MM, carriers of two polymorphic *MIR335* rs3807348 alleles had higher calretinin than carriers of two wild-type alleles (P = 0.027). In this group also carriers of at least one polymorphic *NRF1* rs13241028 C allele had lower calretinin than carriers of two wild-type alleles in the dominant model (P = 0.034), but no significant differences were observed in group of MM patients.

**TABLE 4.** Association of selected SNPs with serum calretinin concentration

**SNP**	**Genotype**	**All subjects**			**Subjects without MM**			**MM patients**		

		**Calretinin (ng/ml) Median (25–75%)**	**P_add_**	**P_dom_**	**Calretinin (ng/ml) Median (25–75%)**	**P_add_**	**P_dom_**	**Calretinin (ng/ml) Median (25–75%)**	**P_add_**	**P_dom_**
***CALB2* rs1862818**	CC	0.18 (0.11–0.34)	0.622	0.422	0.15 (0.09–0.22)	0.751	0.865	0.64 (0.22–1.45)	0.952	0.802
	CT	0.19 (0.11–0.41)			0.16 (0.09–0.24)			0.51 (0.23–1.41)		
	TT	0.18 (0.10–0.37)			0.13 (0.08–0.20)			0.38 (0.21–3.57)		
	CT+TT	0.19 (0.11–0.40)			0.15 (0.09–0.24)			0.48 (0.23–1.43)		
***CALB2* rs889704**	CC	0.19 (0.11–0.37)	0.099	**0.036**	0.15 (0.10–0.23)	0.130	0.069	0.52 (0.25–1.43)	0.508	0.441
	CA	0.17 (0.08–0.27)			0.16 (0.08–0.21)			0.44 (0.14–1.35)		
	AA	0.21 (0.05–0.77)			0.10 (0.02–0.21)			1.07 (0.28–1.84)		
	CA+AA	0.17 (0.08–0.28)			0.14 (0.07–0.21)			0.50 (0.15–1.51)		
***CALB2* rs8063760**	CC	0.18 (0.11–0.38)	0.955	0.770	0.14 (0.09–0.22)	0.382	0.647	0.53 (0.24–1.44)	0.326	0.768
	CT	0.18 (0.12–0.32)			0.16 (0.1–0.24)			0.44 (0.19–1.30)		
	TT	0.21 (0.06–0.51)			0.12 (0.05–0.22)			0.86 (0.50–2.30)		
	CT+TT	0.19 (0.11–0.34)			0.16 (0.09–0.24)			0.51 (0.21–1.43)		
***E2F2* rs2075995**	CC	0.19 (0.10–0.46)	0.512	0.481	0.14 (0.08–0.2)	0.161	0.059	0.72 (0.33–1.45)	0.189	0.117
	CA	0.18 (0.12–0.34)			0.16 (0.1–0.23)			0.53 (0.20–1.48)		
	AA	0.18 (0.10–0.33)			0.14 (0.09–0.24)			0.40 (0.18–0.90)		
	CA+AA	0.18 (0.11–0.34)			0.15 (0.1–0.23)			0.48 (0.20–1.44)		
***MIR335* rs3807348**	GG	0.18 (0.09–0.34)	0.057	0.151	0.14 (0.08–0.2)	**0.027**	0.081	0.44 (0.26–1.43)	0.400	0.978
	GA	0.18 (0.11–0.34)			0.14 (0.09–0.22)	AA ***vs***. GG P = 0.029		0.50 (0.18–1.16)		
	AA	0.21 (0.13–0.39)			0.18 (0.11–0.26)			0.65 (0.27–1.80)		
	GA+AA	0.19 (0.11–0.37)			0.15 (0.1–0.23)			0.52 (0.22–1.44)		
***NRF1* rs13241028**	TT	0.19 (0.12–0.36)	0.272	0.144	0.16 (0.1–0.23)	0.096	**0.034**	0.52 (0.21–1.15)	0.381	0.672
	TC	0.18 (0.10–0.33)			0.14 (0.08–0.21)			0.64 (0.25–1.67)		
	CC	0.17 (0.07–0.36)			0.15 (0.07–0.3)			0.24 (0.07–1.18)		
	TC+CC	0.18 (0.09–0.34)			0.14 (0.08–0.21)			0.46 (0.24–1.53)		
***SEPTIN7* rs3801339**	TT	0.18 (0.11–0.34)	0.403	0.419	0.14 (0.09–0.2)	0.424	0.288	0.35 (0.17–1.05)	0.079	0.080
	TC	0.18 (0.11–0.33)			0.15 (0.09–0.22)			0.51 (0.21–1.23)		
	CC	0.20 (0.11–0.45)			0.16 (0.09–0.25)			0.72 (0.38–1.48)		
	TC+CC	0.19 (0.11–0.37)			0.15 (0.09–0.23)			0.64 (0.26–1.45)		

A = adenine; Add = additive model, calculated using Kruskal-Wallis test; C = cytosine; Dom = dominant model, calculated using Mann-Whitney test; G = guanine; MM = malignant mesothelioma, SNP = single nucleotide polymorphism, T = thymine

Association of selected SNPs with serum calretinin concentration in subjects with asbestosis, subjects with pleural plaques and subjects without disease is shown in Supplementary Table 3. In subjects without asbestos-related disease, carriers of at least one polymorphic *CALB2* rs889704 A allele had lower calretinin than carriers of two wild-type alleles in the additive model (P = 0.014) and dominant model (P = 0.004), but no significant differences were observed in subjects with pleural plaques (P_add_ = 0.060, P_dom_ = 0.300) and subjects with asbestosis (P_add_ = 0.290, P_dom_ = 0.279). In subjects with pleural plaques, carriers of at least one polymorphic *NRF1* rs13241028 C allele had lower calretinin than carriers of two wild-type alleles in the dominant model (P = 0.025). In subjects with asbestosis, carriers of at least one polymorphic *E2F2* rs2075995 A allele had higher calretinin than carriers of two wild-type alleles in the additive model (P = 0.049) and dominant model (P = 0.017). With ROC curve analysis, we compared serum calretinin levels in MM patients with all other subjects according to individual genotypes for SNPs, which affected calretinin levels in at least one group. In almost all groups, calretinin concentration could significantly discriminate between MM patients and other subjects with good sensitivity and specificity (Table 5). Optimal calretinin cut off values differed according to genotype, even though the differences were small. For *CALB2* rs889704, lower cut off was observed in carriers of two polymorphic alleles (0.21 *vs*. 0.32 ng/ml). For *E2F2* rs2075995, higher cut off was observed in carriers of two polymorphic alleles (0.33 *vs*. 0.26 ng/ml). For *MIR335* rs3807348, higher cut off was observed in carriers of two polymorphic alleles (0.35 *vs*. 0.29 ng/ml). For *NRF1* rs13241028, lower cut off was observed in carriers of at least one polymorphic alleles (0.23 *vs*. 0.32 ng/ml) (Table 5).

**TABLE 5.** Receiver operating characteristic (ROC) curve analysis according to individual genotypes for selected single nucleotide polymorphisms: comparison of malignant mesothelioma (MM) patients with all other subjects

**SNP**	**Genotype**	**AUC (95% CI)**	**P**	**Calretinin cut-off (ng/ml)^1^**	**Sensitivity**	**Specificity**
Overall analysis in the whole group	/	0.825 (0.781–0.868)	< 0.001	0.32	0.681	0.887
***CALB2* rs889704**	CC	0.831 (0.782–0.880)	< 0.001	0.32	0.695	0.876
	CA	0.779 (0.667–0.891)	< 0.001	0.31	0.607	0.935
	AA^2^	0.958 (0.837–1.000)	0.019	0.21	1.000	0.833
	CA+AA	0.801 (0.702–0.901)	< 0.001	0.31	0.625	0.940
***E2F2* rs2075995**	CC	0.906 (0.845–0.968)	< 0.001	0.26	0.810	0.903
	CA	0.803 (0.736–0.869)	< 0.001	0.32	0.671	0.888
	AA	0.781 (0.686–0.876)	< 0.001	0.33	0.615	0.877
	CA+AA	0.797 (0.742–0.851)	< 0.001	0.32	0.653	0.881
***MIR335* rs3807348**	GG	0.853 (0.766–0.940)	< 0.001	0.29	0.757	0.872
	GA	0.803 (0.739–0.867)	< 0.001	0.32	0.643	0.892
	AA	0.845 (0.765–0.925)	< 0.001	0.35	0.738	0.881
	GA+AA	0.815 (0.764–0.866)	< 0.001	0.32	0.675	0.881
***NRF1* rs13241028**	TT	0.812 (0.754–0.871)	< 0.001	0.32	0.693	0.884
	TC	0.868 (0.804–0.931)	< 0.001	0.23	0.818	0.798
	CC^3^	0.664 (0.406–0.922)	0.203	0.18	0.714	0.700
	TC+CC	0.842 (0.777–0.907)	< 0.001	0.23	0.790	0.785

1 Cut-off with the highest sum of sensitivity and specificity;

2 based on 10 subjects,

3 based on 27 subjects.

A = adenine; AUC = area under the curve; C = cytosine; G = guanine; SNP = single nucleotide polymorphism; T = thymine

### Haplotype analysis

Analysis of *CALB2* haplotypes identified eight SNP combinations. The most common haplotype was CCC with predicted frequency 0.449, followed by TCC (0.261), CCT (0.167), CAT (0.060), CAC (0.045), TCT (0.009), TAC (0.007) and TAT (0.003). Haplotype TCC was more common in MM patients, but the association was not statistically significant (P = 0.061, Table 6). *CALB2* haplotypes were not associated with serum calretinin concentrations (Table 6).

**TABLE 6.** Association of *CALB2* haplotypes with malignant mesothelioma (MM) susceptibility and serum calretinin concentration

**Haplotype**	**Subjects without MM Predicted frequency**	**MM patients Predicted frequency**	**OR (95% CI)**	**P**	**OR (95% CI)_adj_**	**P_adj_**	**Serum calretinin concentration P**
CCC	0.457	0.431	Reference		Reference		
TCC	0.245	0.294	1.26 (0.0–991.60)	0.061	1.26 (0.97–1.64)	0.084	0.272
CCT	0.176	0.147	0.88 (0.65–1.20)	0.415	0.94 (0.66–1.34)	0.731	0.125
CAT	0.058	0.066	1.21 (0.77–1.89)	0.408	1.08 (0.64–1.81)	0.782	0.731
CAC	0.045	0.047	1.11 (0.64–1.91)	0.713	0.99 (0.55–1.79)	0.974	0.852

The SNPs are ordered from the 5′- to 3′-end as follows: rs1862818, rs889704, rs8063760.

A = adenine; Adj = adjusted for age, C = cytosine; CI = confidence interval; MM = malignant mesothelioma; OR = odds ratio; SNP = single nucleotide polymorphism; T = thymine

## Discussion

In the present study, we evaluated the role of genetic variability in *CALB2* and its regulatory miRNA and transcription factors genes with serum calretinin levels and MM susceptibility. Genetic variability of *CALB2* was associated with calretinin concentration, but not with MM susceptibility. For SNPs in genes regulating calretinin expression, differences in genotype frequencies among MM and other subjects were also observed. Additionally, genetic factors influenced optimal serum calretinin cut off values differentiating MM patients from other asbestos-exposed subjects.

Using bioinformatic analysis, we identified seven common putatively functional SNPs that could affect calretinin expression: three SNPs in *CALB2* gene, one SNP in transcription factor *E2F2*, one SNP in transcription factor *NRF1*, one SNP in transcription factor *SEPTIN7* and one SNP in miRNA *MIR335*. In previous studies, demographic and clinical factors such as sex and renal function affecting plasma or serum calretinin concentration in asbestos-related diseases were already identified^21,22,41^, but the role of genetic variability is largely unexplored.

Among *CALB2* SNPs investigated in our study, *CALB2* rs1862818 and *CALB2* rs889704 may influence transcription factor binding, while *CALB2* rs8063760 may influence miRNA binding. In our study, *CALB2* rs889704 was associated with lower serum calretinin levels in all subjects and subjects without asbestos-related diseases, while there was no association in patients with MM. None of the selected *CALB2* SNPs or haplotypes were significantly associated with MM susceptibility. To the best of our knowledge, the functional role of *CALB2* SNPs and their association with asbestos-related diseases was not investigated yet. However, one intronic SNP in *CALB2* was previously associated with calretinin expression in tumor cell lines and the development of colon cancer, but no association with lung cancer was observed.^30^ Data on *CALB2* genetic variability are therefore lacking and further studies are needed to evaluate its role in MM and serum calretinin levels.

Three important transcription factors were previously associated with regulation of calretinin.^23,24^ E2F2 is a transcription factor that binds to *CALB2* promoter and was associated with calretinin expression in mesothelioma cell lines.^23^ In our study, *E2F2* rs2075995 was associated with decreased MM risk. When comparing MM patients to only subjects without disease, the association remained significant even after taking into account the age of the subjects. *E2F2* rs2075995 was also associated with higher serum calretinin level among subjects with asbestosis. E2F2 has an important role in the regulation of cell cycle, but also affects other important processes such as cell proliferation, apoptosis and inflammation.^42^ In cancer, it was mostly associated with promoting tumor progression in various malignancies, including lung cancer.^42^ E2F2 could also contribute to the cell cycle-dependent differences observed for calretinin expression.^23^ *E2F2* rs2075995 is a nonsynonymous SNP and may influence splicing. So far, *E2F2* rs2075995 was only evaluated in patients with colorectal cancer, where no association with cancer risk was observed.^43,44^ However, no studies evaluated the association of *E2F2* rs2075995 with MM. Still, the E2F gene family was often associated with different types of cancer. Several other *E2F2* polymorphisms were associated with oral and oropharyngeal squamous cell carcinoma risk and might also affect the course of the disease.^45^ Combinations of different *E2F2* gene SNPs were proposed as a risk factor for squamous cell carcinoma of the head and neck.^46^ The *E2F2* gene was also associated with ovarian cancer risk.^47^ Additionally, *E2F2* genetic variability was proposed as recurrence biomarker in squamous cell carcinoma of the oropharynx.^48^ Among other *E2F2* SNPs, rs3218211 was in very high LD with rs2075995 investigated in our study. *E2F2* rs3218211 was associated with T stage in oral and oropharyngeal squamous cell carcinoma and decreased head and neck squamous cell carcinoma risk, consistent with our results.^45,46^ Taken together, this suggests further studies regarding the role of *E2F2* genetic variability in asbestos-related diseases and its association with calretinin are needed.

The second important calretinin-related transcription factor is NRF-1. It binds to *CALB2* promoter and might be important for the transcriptional control of calretinin expression in MM.^23^ In our study, *NRF1* rs13241028 was associated with lower serum calretinin level in subjects without MM, but it was not associated with MM susceptibility. NRF-1 regulates expression of various genes involved in oxidative phosphorylation, mitochondrial biogenesis and other mitochondrial processes, including transcription of mitochondrial DNA.^49^ Additionally, NRF-1 can modify different aspects of carcinogenesis, including proliferation, invasion, and apoptosis.^50^ *NRF1* rs13241028 may influence miRNA binding.^51^ So far, *NRF1* genetic variability has been associated primarily with increased susceptibility to diabetes.^52,53^ *NRF1* has also been associated with epithelial ovarian cancer risk.^54^ Further studies are needed to better evaluate the role of NRF-1 and its genetic variability in asbestos-related diseases.

Septin 7 has also been identified as a factor that binds to the *CALB2* promoter region, resulting in decreased calretinin expression in mesothelioma cell lines.^24^ Septin 7 is a GTP-binding protein that is involved in cytokinesis, cytoskeleton organization and other cellular processes.^24,55^ It was also implicated in calcium homeostasis.^56^ Several studies also reported that septin 7 plays an important role in cancer development, especially glioma.^55,56^ In our study, *SEPTIN7* rs3801339 was not significantly associated with MM susceptibility or with serum calretinin levels. The functional role of *SEPTIN7* rs3801339 is not yet understood: it was previously classified as a non-synonymous variant, while it is now described as a genic downstream transcript variant. Interestingly, *SEPTIN7* rs1143149 in moderate LD with rs3801339 was proposed as a risk factor for the development of non-small cell lung cancer and was associated with shorter survival in long-term smokers.^55^ *SEPTIN7* was often mutated in breast ductal carcinoma *in situ* cell lines and these mutations might participate in the progression of breast ductal carcinoma.^57^ Recent studies therefore suggest that *SEPTIN7* variability may play a role in some cancers, but it was not an important risk factor in asbestos-related diseases in our study.

MiRNAs affect gene expression on the post-transcriptional level and are often deregulated in cancer.^58^ Among miRNAs predicted to modify calretinin expression, common polymorphisms were only described for miR-335. In our study, carriers of two polymorphic *MIR335* rs3807348 alleles were more likely to develop MM compared to subjects with asbestosis, even after adjustment for age. *MIR335* rs3807348 was also associated with serum calretinin level in subjects without MM. MiR-335 can modulate cell proliferation, apoptosis, migration and invasion through various signaling pathways. It mostly acts as a tumor suppressor and is downregulated in different cancer types.^58^ *MIR335* rs3807348 may influence transcription factor binding, but its role has not been experimentally confirmed. To date, no research has been done on the association of rs3807348 with MM. *MIR335* rs3807348 was not associated with breast cancer risk in a previous study^59^, but more studies would be needed in this field.

As several genetic factors were associated with calretinin, we also evaluated how these factors influence serum calretinin cut off values. We found that four SNPs, *CALB2* rs889704, *E2F2* rs2075995, *MIR335* rs3807348, and *NRF1* rs13241028 could be used to fine tune serum calretinin cut off values predicting MM. Calretinin as a biomarker could thus have higher sensitivity and specificity in individuals with known genetic variability. Similar results were observed for mesothelin, where predictive value was improved when taking into account polymorphisms located in 5′ UTR and 3′ UTR of the *MSLN* gene.27–29 In the future, combination of clinical and genetic factors could thus help guide calretinin cut-off values and decrease false negative or positive results.

This is the first study to show that genetic factors can affect serum calretinin levels and that accounting for these genetic factors may improve the predictive value of serum calretinin. We have also shown that genetic factors associated with calretinin may play a role in the development of mesothelioma. A limitation of our study is that we only had serum calretinin concentrations available for a subgroup of participants included in the study. On the other hand, we performed a comprehensive analysis of the factors that could affect calretinin expression using literature review and detailed bioinformatics analysis. Genetic variability was evaluated in a large cohort, which gives additional power to the study. However, other polymorphisms in the investigated genes could also affect calretinin concentration and other factors could affect calretinin regulation. In the future, further studies in this field and validation of these results in an independent population are needed.

## Conclusions

The present study showed that genetic variability in *CALB2* gene and genes coding for transcription factors and miRNAs that regulate calretinin expression could contribute to interindividual differences in serum calretinin levels in MM patients or asbestos-exposed subjects. These results could contribute to a better understanding of calretinin regulation and could potentially contribute to an earlier diagnosis of MM.

## Supplementary Material

Supplementary Material Details
