# Supplementary material for: The association of genetic factors with serum calretinin levels in asbestos-related diseases
Source: Radiol Oncol. 2023 Nov 30;57(4):473–86. doi: 10.2478/raon-2023-0061 (PMC10690752; doi:10.2478/raon-2023-0061)
Supplement: Supplementary file 1 — Supplementary Material Details [file raon-2023-0061_sm.pdf]

The association of genetic factors with serum calretinin levels in asbestos−related diseases

Cita Zupanc, Alenka Franko, Danijela Strbac, Viljem Kovac, Vita Dolzan, Katja Goricar

doi: 10.2478/raon-2023-0061

SUPPLEMENTARY TABLE 1. Genotype frequencies of investigated single nucleotide polymorphisms among subjects without asbestos-related diseases, subjects with pleural plaques and subjects with asbestosis

Gene	SNP	Genotype	No disease (N = 83) N (%)	Pleural plaques (N = 380) N (%)	Asbestosis (N = 153) N (%)
CALB2	rs1862818	CC	45 (54.2)	211 (55.5)	84 (54.9)
		CT	31 (37.3)	138 (36.3)	57 (37.3)
		TT	7 (8.4)	31 (8.2)	12 (7.8)
CALB2	rs889704	CC	61 (73.5)	302 (79.7) [1]	122 (79.7)
		CA	20 (24.1)	72 (19.0)	29 (19.0)
		AA	2 (2.4)	5 (1.3)	2 (1.3)
CALB2	rs8063760	CC	50 (61.0) [1]	211 (55.7) [1]	91 (59.5)
		CT	26 (31.7)	141 (37.2)	55 (35.9)
		TT	6 (7.3)	27 (7.1)	7 (4.6)
E2F2	rs2075995	CC	10 (12.0)	79 (20.8)	28 (18.3)
		CA	45 (54.2)	189 (49.7)	85 (55.6)
		AA	28 (33.7)	112 (29.5)	40 (26.1)
MIR335	rs3807348	GG	19 (23.2) [1]	89 (23.5) [1]	50 (32.9) [1]
		GA	45 (54.9)	191 (50.4)	71 (46.7)
		AA	18 (22.0)	99 (26.1)	31 (20.4)
NRF1	rs13241028	TT	48 (57.8)	237 (62.4)	89 (58.2)
		TC	34 (41.0)	118 (31.1)	58 (37.9)
		CC	1 (1.2)	25 (6.6)	6 (3.9)
SEPTIN7	rs3801339	TT	14 (16.9)	71 (18.7)	24 (15.7)
		TC	33 (39.8)	166 (43.7)	67 (43.8)
		CC	36 (43.4)	143 (37.6)	62 (40.5)

Number of missing data is presented in [] brackets. SNP = single nucleotide polymorphism.
A = adenine; C = cytosine; G = guanine; SNP = single nucleotide polymorphisms; T = thymine

SUPPLEMENTARY TABLE 2. Association of investigated single nucleotide polymorphisms (SNPs) with malignant mesothelioma (MM) susceptibility

		Comparison of MM patients with subjects without any disease				Comparison of MM patients with subjects with pleural plaques				Comparison of MM patients with subjects with asbestosis			
SNP	Genotype	OR (95% CI)	P	OR (95% CI)$_{adj}$	P_{adj}	OR (95% CI)	P	OR (95% CI)$_{adj}$	P_{adj}	OR (95% CI)	P	OR (95% CI)$_{adj}$	P_{adj}
CALB2 rs1862818	CC	Reference		Reference		Reference		Reference		Reference		Reference	
	CT	1.25 (0.75–2.10)	0.394	1.16 (0.65–2.07)	0.619	1.32 (0.95–1.83)	0.094	1.27 (0.88–1.82)	0.204	1.27 (0.84–1.93)	0.256	1.39 (0.90–2.15)	0.143
	TT	1.34 (0.55–3.27)	0.519	1.45 (0.52–4.02)	0.479	1.42 (0.82–2.46)	0.211	1.29 (0.69–2.38)	0.425	1.46 (0.71–3.02)	0.306	1.45 (0.67–3.10)	0.343
	CT+TT	1.27 (0.78–2.07)	0.340	1.21 (0.70–2.09)	0.503	1.34 (0.98–1.82)	0.063	1.27 (0.90–1.79)	0.174	1.30 (0.88–1.93)	0.185	1.40 (0.92–2.12)	0.113
CALB2 rs889704	CC	Reference		Reference		Reference		Reference		Reference		Reference	
	CA	0.83 (0.47–1.49)	0.540	0.89 (0.46–1.71)	0.719	1.15 (0.78–1.68)	0.481	1.03 (0.67–1.58)	0.896	1.15 (0.70–1.89)	0.578	1.10 (0.65–1.86)	0.714
	AA	0.55 (0.10–3.06)	0.492	0.23 (0.03–1.64)	0.143	1.08 (0.29–4.08)	0.906	0.53 (0.12–2.29)	0.397	1.09 (0.20–6.06)	0.918	0.60 (0.10–3.42)	0.564
	CA+AA	0.81 (0.46–1.42)	0.456	0.81 (0.43–1.53)	0.512	1.14 (0.79–1.66)	0.482	0.99 (0.65–1.50)	0.949	1.15 (0.71–1.86)	0.576	1.06 (0.64–1.77)	0.816
CALB2 rs8063760	CC	Reference		Reference		Reference		Reference		Reference		Reference	
	CT	1.07 (0.62–1.82)	0.815	0.97 (0.53–1.76)	0.918	0.83 (0.60–1.15)	0.263	0.86 (0.60–1.24)	0.420	0.92 (0.60–1.39)	0.684	0.88 (0.57–1.36)	0.571
	TT	0.76 (0.28–2.05)	0.590	0.56 (0.18–1.74)	0.321	0.71 (0.37–1.37)	0.311	0.65 (0.31–1.35)	0.248	1.19 (0.47–2.99)	0.714	1.05 (0.40–2.75)	0.928
	CT+TT	1.01 (0.61–1.67)	0.972	0.89 (0.51–1.56)	0.688	0.81 (0.59–1.11)	0.187	0.82 (0.58–1.17)	0.279	0.95 (0.64–1.41)	0.793	0.90 (0.59–1.37)	0.624

E2F2 rs2075995	CC	Reference		Reference		Reference		Reference		Reference		Reference	
	CA	0.47 (0.23–0.99)	**0.048**	0.47 (0.21–1.07)	0.072	0.89 (0.60–1.31)	0.554	1.00 (0.64–1.54)	0.982	0.70 (0.42–1.17)	0.175	0.71 (0.41–1.21)	0.210
	AA	0.35 (0.16–0.78)	**0.010**	0.35 (0.14–0.84)	**0.019**	0.70 (0.45–1.08)	0.105	0.73 (0.45–1.20)	0.214	0.69 (0.38–1.24)	0.215	0.72 (0.39–1.33)	0.297
	CA+AA	0.43 (0.21–0.87)	**0.019**	0.43 (0.19–0.94)	**0.033**	0.82 (0.57–1.18)	0.280	0.89 (0.59–1.35)	0.596	0.70 (0.43–1.14)	0.150	0.71 (0.42–1.19)	0.197
MIR335 rs3807348	GG	Reference		Reference		Reference		Reference		Reference		Reference	
	GA	0.84 (0.46–1.54)	0.570	0.85 (0.43–1.67)	0.642	0.93 (0.63–1.36)	0.690	0.88 (0.57–1.35)	0.546	1.40 (0.88–2.22)	0.155	1.47 (0.91–2.39)	0.118
	AA	1.19 (0.58–2.45)	0.634	1.20 (0.54–2.65)	0.659	1.01 (0.66–1.56)	0.947	0.98 (0.60–1.58)	0.925	1.82 (1.05–3.16)	**0.033**	1.96 (1.10–3.50)	**0.022**
	GA+AA	0.94 (0.53–1.68)	0.832	0.95 (0.50–1.81)	0.881	0.96 (0.67–1.37)	0.805	0.91 (0.61–1.36)	0.651	1.53 (0.99–2.35)	0.055	1.62 (1.03–2.55)	**0.037**
NRF1 rs13241028	TT	Reference		Reference		Reference		Reference		Reference		Reference	
	TC	0.84 (0.51–1.39)	0.498	0.89 (0.51–1.57)	0.694	1.20 (0.86–1.66)	0.287	1.30 (0.90–1.88)	0.165	0.91 (0.61–1.38)	0.666	0.89 (0.58–1.36)	0.583
	CC	3.33 (0.42–26.25)	0.254	4.83 (0.57–40.82)	0.149	0.66 (0.32–1.35)	0.251	0.73 (0.33–1.61)	0.440	1.03 (0.37–2.83)	0.956	1.39 (0.49–3.96)	0.537
	TC+CC	0.91 (0.56–1.50)	0.714	1.00 (0.58–1.75)	0.991	1.10 (0.80–1.51)	0.546	1.20 (0.84–1.71)	0.310	0.92 (0.62–1.38)	0.699	0.93 (0.61–1.41)	0.731
SEPTIN7 rs3801339	TT	Reference		Reference		Reference		Reference		Reference		Reference	
	TC	1.04 (0.52–2.10)	0.910	0.89 (0.40–2.00)	0.783	1.05 (0.69–1.60)	0.820	1.11 (0.69–1.78)	0.660	0.88 (0.50–1.54)	0.653	0.83 (0.46–1.49)	0.527
	CC	0.69 (0.34–1.40)	0.304	0.51 (0.23–1.16)	0.107	0.88 (0.57–1.37)	0.582	0.84 (0.52–1.37)	0.495	0.69 (0.39–1.23)	0.206	0.64 (0.35–1.17)	0.144
	TC+CC	0.86 (0.45–1.64)	0.646	0.69 (0.33–1.46)	0.334	0.97 (0.66–1.44)	0.893	0.98 (0.63–1.52)	0.936	0.79 (0.47–1.33)	0.375	0.73 (0.42–1.28)	0.276

A = adenine; Adj = adjusted for age; C = cytosine; CI = confidence interval; G = guanine; OR = odds ratio; SNP = single nucleotide polymorphism; T = thymine

SUPPLEMENTARY TABLE 3. Association of selected single nucleotide polymorphisms with serum calretinin concentration

		Subject without asbestos-related disease			Subjects with pleural plaques			Subjects with asbestosis		
**SNP**	**Genotype**	**Calretinin (ng/ml) Median (25–75%)**	**P_{add}**	**P_{dom}**	**Calretinin (ng/ml) Median (25–75%)**	**P_{add}**	**P_{dom}**	**Calretinin (ng/ml) Median (25–75%)**	**P_{add}**	**P_{dom}**
CALB2 rs1862818	CC	0.12 (0.07–0.21)	0.533	0.520	0.17 (0.12–0.25)	0.855	0.790	0.13 (0.08–0.20)	0.996	0.943
	CT	0.12 (0.06–0.19)			0.18 (0.11–0.24)			0.13 (0.06–0.23)		
	TT	0.08 (0.07–0.12)			0.16 (0.11–0.28)			0.13 (0.09–0.20)		
	CT+TT	0.11 (0.07–0.18)			0.18 (0.11–0.24)			0.13 (0.07–0.21)		
CALB2 rs889704	CC	0.12 (0.09–0.22)	**0.014** CA vs. CC P = 0.012	**0.004**	0.18 (0.12–0.26)	0.060	0.300	0.12 (0.08–0.19)	0.290	0.279
	CA	0.07 (0.04–0.12)			0.18 (0.09–0.23)			0.16 (0.07–0.23)		
	AA	0.10 (0.06–0.10)			0.02 (0.00–0.02)			0.22 (0.21–0.22)		
	CA+AA	0.07 (0.04–0.12)			0.18 (0.08–0.23)			0.16 (0.08–0.24)		
CALB2 rs8063760	CC	0.12 (0.07–0.19)	0.214	0.838	0.18 (0.11–0.25)	0.312	0.862	0.13 (0.07–0.19)	0.179	0.350
	CT	0.12 (0.09–0.23)			0.18 (0.13–0.25)			0.13 (0.08–0.21)		
	TT	0.06 (0.05–0.12)			0.14 (0.00–0.22)			0.19 (0.1–0.34)		
	CT+TT	0.11 (0.08–0.22)			0.17 (0.12–0.25)			0.14 (0.08–0.21)		
E2F2 rs2075995	CC	0.07 (0.04–0.12)	0.171	0.064	0.18 (0.13–0.23)	0.959	0.860	0.08 (0.06–0.14)	**0.049** CA vs. CC P = 0.042	**0.017**
	CA	0.12 (0.08–0.18)			0.18 (0.11–0.26)			0.15 (0.1–0.21)		
	AA	0.12 (0.07–0.22)			0.18 (0.11–0.26)			0.13 (0.07–0.24)		
	CA+AA	0.12 (0.07–0.20)			0.18 (0.11–0.26)			0.14 (0.09–0.21)		

MIR335 rs3807348	GG	0.07 (0.04–0.16)	0.053	0.066	0.18 (0.13–0.24)	0.813	0.897	0.13 (0.06–0.17)	0.248	0.150
	GA	0.12 (0.07–0.17)			0.17 (0.11–0.25)			0.13 (0.08–0.21)		
	AA	0.16 (0.10–0.29)			0.18 (0.13–0.28)			0.17 (0.09–0.26)		
	GA+AA	0.12 (0.07–0.21)			0.18 (0.11–0.25)			0.13 (0.09–0.23)		
NRF1 rs13241028	TT	0.12 (0.07–0.18)	0.677	0.962	0.19 (0.13–0.26)	0.069	**0.025**	0.13 (0.09–0.19)	0.769	0.482
	TC	0.12 (0.06–0.22)			0.14 (0.10–0.21)			0.12 (0.05–0.22)		
	CC	0.07[1]			0.16 (0.05–0.35)			0.10 (0.08–0.10)		
	TC+CC	0.12 (0.06–0.22)			0.15 (0.09–0.22)			0.11 (0.06–0.21)		
SEPTIN7 rs3801339	TT	0.15 (0.09–0.19)	0.463	0.487	0.17 (0.09–0.23)	0.222	0.182	0.11 (0.07–0.15)	0.718	0.428
	TC	0.12 (0.08–0.21)			0.17 (0.12–0.24)			0.13 (0.08–0.19)		
	CC	0.10 (0.06–0.18)			0.19 (0.13–0.29)			0.13 (0.07–0.25)		
	TC+CC	0.11 (0.07–0.20)			0.18 (0.13–0.25)			0.13 (0.08–0.21)		

[1] Only one subject with this genotype. A = adenine; Add = additive model, calculated using Kruskal-Wallis test; C = cytosine; Dom = dominant model, calculated using Mann-Whitney test; G = guanine; SNP = single nucleotide polymorphism; T = thymine
